# Harnessing the Potential of Roots of Traditional Power Plant: *Ocimum*

**DOI:** 10.3389/fpls.2021.765024

**Published:** 2021-11-01

**Authors:** Vibha Pandey, Ravi Kant Swami, Alka Narula

**Affiliations:** Department of Biotechnology, School of Chemical and Life Sciences, Jamia Hamdard, New Delhi, India

**Keywords:** root, hairy roots, rhizosphere, medicinal, metabolites, *Agrobacterium rhizogenes*, *Ocimum*

## Abstract

Genus *Ocimum* of Labiatae is well known in all traditional medicinal systems like Ayurveda, Unani, Siddha, and Homeopathy. The pharmaceutical activities of different species of *Ocimum* attributed to all plant parts. Roots are the most significant vital organ of the plant, as they absorb water and nutrients from soil and transport to aerial parts of the plants. Roots of *Ocimum* were found helpful with free-radical scavenging activity to improve physical and mental strength as well as to treat diabetes, malaria, and liver problems. Antibacterial activity of *Ocimum* roots and its main component, rosmarinic acid, is very beneficial to protect against several human pathogens, including bacteria and viruses. Being so important in every way, roots of *Ocimum* need healthy rhizosphere. Bacteria, fungi, nematodes, types of soil, fungicide, pesticides, salt, radioactive elements, as well as heavy metal contaminations, affect roots and overall growth of *Ocimum* in positive or negative ways. Each component of rhizosphere (natural, treatment or contamination) affects the roots, which highlights current ecological scenario to discover biosafe and more productive approaches. For such prestigious organ of *Ocimum*, development of *in vitro* root cultures and hairy root cultures assists to reduce the efforts and timing of the traditional cultivation process along with elimination of negative factors in rhizosphere. Different strains of *Agrobacterium rhizogenes*, various media compositions, as well as discrete treatments, like elicitors, on nonidentical species or cultivars of *Ocimum* boost the root induction, biomass, and accumulation of phytoceuticals differently. Hairy roots and *in vitro* roots of *Ocimum* accumulate higher quantity of therapeutic metabolites. These metabolites include several phenolics (like rosmarinic acid, 3-hydroxybenzoic acid, m-coumaric acid, p-coumaric acid, caffeic acid, ferulic acid, vanillic acid, chicoric acid, and lithospermic acid), triterpenes (such as betulinic acid, 3-epimaslinic acid, alphitolic acid, euscaphic acids, oleanolic acid, and ursolic acid) as well as flavonoids (flavones, flavonols, and dihydroflavonols). This review highlights pharmaceutical applications of *Ocimum* roots, a great deal of rhizosphere components and *in vitro* culturing techniques to enhance biomass as well as chief phytoceuticals.

## Introduction

Genus *Ocimum* (Family Labiatae) is among the most important plants with therapeutic potential due to its worldwide cultivation, including, Australia, Malaysia, Africa (east, north, and west), India, China, Taiwan, and several Arab countries (Mondal et al., 2009; Baliga et al., 2013; Kulkarni and Adavirao, 2018). *O. sanctum* L. (or *Ocimum tenuiflorum*), *O. basilicum*, *O. canum*, *O. gratissimum*, *O. americanum*, *O. camphora*, *O. kilimandscharicum*, and *O. micranthum* are explored medicinally important species of *Ocimum* (Pattanayak et al., 2010; Rahman et al., 2011; Kulkarni and Adavirao, 2018; Das et al., 2020; Singh et al., 2020).

All parts (root, stem, leaves, flower, and seeds), as well as whole the plant of *Ocimum*, have been recommended in traditional medicinal systems. Potential therapeutic properties of *Ocimum* include adaptogenic, anabolic, analgesic, antiaging, antiallergic, antiarthritic, anticancer, anticataract, anticoagulant, anticonvulsant, antidiabetic, antidiuretic, antiemetic, antifertility, antihistaminic, anti-inflammatory, antilipidemic, antimicrobial, antioxidant, antipyretic, antispasmodic, antistress, antiulcer, cardioprotective, chemopreventive, diaphoretic, hepatoprotective, hypolipidemic, immunomodulatory, immune stimulant, larvicidal, mosquito repellent, neuroprotective, radioprotective, and other activities (Gupta et al., 2002; Prakash and Gupta, 2005; Singh et al., 2007; Rahman et al., 2011; Baliga et al., 2013; Jamshidi and Cohen, 2017; Wakchaure et al., 2017; Kulkarni and Adavirao, 2018; Gautam et al., 2020; Kurepa and Smalle, 2021).

Mostly, aerial parts of *Ocimum* have been reported with active biological components, including flavonoids, saponins, tannins, and triterpenoids in stems and leaves (Pattanayak et al., 2010), as well as aldehydes, phenol, phenylpropanoids, and terpenes in seeds (Mondal et al., 2009). A wide range of metabolites has been identified from *Ocimum*, including apigenin, apigenin-7-O-glucuronide, α-terpineol, β-bisabolene, bornyl acetate, camphene, campesterol, carnosic acid, carvacrol, caryophyllene, circimaritin, cirsilineol, α-elemene, eugenol, euginal, isothymusin, isothymonin, linalool, limatrol, luteolin, luteolin-7-O glucuronide, methyl chavicol, molludistin, myretenal, α- and β-pinenes, oleanlic acid, orientin, rosmarinic acid, sitosterol, stigmasterol, ursolic acid, and vicenin (Gupta et al., 2002; Pattanayak et al., 2010; Shilpa et al., 2010; Baliga et al., 2013; Vyas and Mukhopadhyay, 2014; Wakchaure et al., 2017; Chaudhary et al., 2020; Singh et al., 2020).

*Ocimum sanctum*, which belongs to Ayurvedic and Unani medicinal system, is the most studied indigenous plant of the Indian subcontinent. The plant is also called as Holy Tulsi and known to have two varieties, Krishna or Hari Tulsi and Ram or Shri Tulsi, with similar biochemical and therapeutic properties. The importance of tulsi made it the “queen of herbs,” “elixir of life,” “matchless one,” “incomparable one,” “a herb for all reasons,” or “mother medicine of nature” (Wakchaure et al., 2017; Kulkarni and Adavirao, 2018; Gautam et al., 2020; Nadar et al., 2020; Singh et al., 2020; Kurepa and Smalle, 2021). *O. sanctum* is an erect, branched, annual herb with a square stem, which grows up to 18 inches (75 cm) into low bushes and has specific aroma (Gupta et al., 2002; Prakash and Gupta, 2005; Mondal et al., 2009; Pattanayak et al., 2010; Shilpa et al., 2010; Rahman et al., 2011; Kulkarni and Adavirao, 2018). Therapeutic metabolic of *O. sanctum* is responsible for the defense mechanism of the plant (Vyas and Mukhopadhyay, 2014). Antimicrobial properties of Tulsi have been utilized and available in the form of hand sanitizer, mouthwash, preservatives, and water purifier (Cohen, 2014). *O. basilicum*, which is also called sweet basil, is another utmost studied *Ocimum* species known for its pharmaceutical significance and the responsible metabolites (Marzouk, 2009).

Researchers have mentioned all parts of the *Ocimum* with pharmaceutical significance. Most studies focused on therapeutic meaning of aerial parts of the plants, especially related to essential oil, eugenol, and rosmarinic acid. Roots of the plant also possess same medicinal importance as aerial parts are enriched with considered amount and variety of phytoceuticals.

**FIGURE 1:**
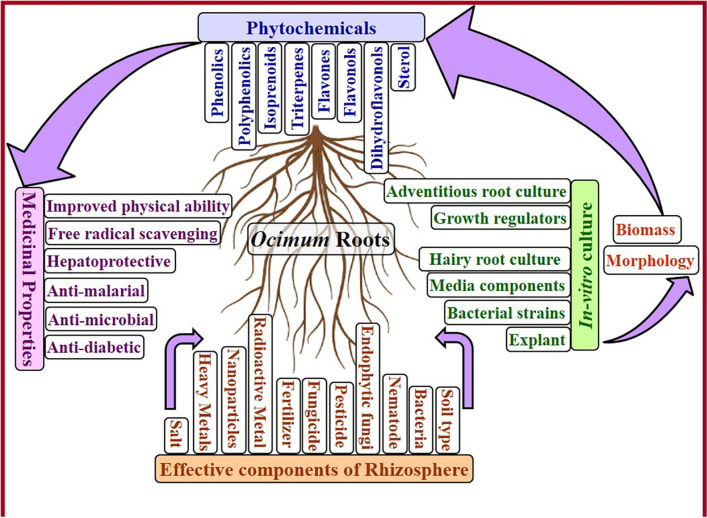
Factors affecting *Ocimum* root growth and its phytoconstituents.

This review highlights the importance of *Ocimum* roots by bringing available research (Figure 1). Because of emphasized therapeutical benefits of *Ocimum* roots, a systematic learning of its root and rhizosphere is a prerequisite. Due to the significance of rhizosphere and increasing contamination of soil and water, a summarized effect of soil type, microbes, nematodes, agricultural treatments, and contaminants (from industries) in rhizosphere on roots and growth of the whole plant of *Ocimum* is discussed in detail. Variable rhizosphere of *Ocimum* roots under *in vivo* conditions affects nutritional uptake, plant growth, and its medicinal properties, which can be overcome by *in vitro* cultures. In order to establish productive root culture, optimization of basal media, plant growth hormone, *Agrobacterium rhizogenes* strain, and elicitor treatment is vital; thus, it is briefly discussed in this review.

## Pharmacological Roots

Like other parts of plant, roots of *Ocimum sp*. conquer the therapeutic potential due to metabolic content of roots. Most studies used different types of root extracts to investigate curative activity of roots of *O. sanctum*, *O. basilicum*, *O. canum*, and *O. campechianum*. Detailed information on species-specific analysis for particular disease or pathogen is summarized in Table 1.

**TABLE 1:** Medicinal properties of different extracts and purified components of *Ocimum species.*

Plant	Activities	Treatment with/Analysis of	Findings	References
*O. sanctum*	Against disabled physical activity	Methanolic root extract	Decrease in mean swimming time	Maity et al., 2000
		Petroleum ether extract of roots	Decrease in mean swimming time, number of errors to reach the destination, sleeping time	Maity et al., 2003
		Ethyl acetate fraction of methanolic root extract	Inhibition of : Increased paw volume (anti-inflammatory); painful sensation, number of writhing, licking time (analgesic); temperature (anti-pyretic)	Kumar et al., 2015
	Anti-plasmodial activity	Hexane, chloroform, ethyl acetate, acetone and methanol extract of roots	Inhibition in growth of *Plasmodium falciparum* (IC50 value of 11.47 and 14.04 μg/ml)	Venkatesalu et al., 2012
	Radical scavenging activity	Ethyl acetate extract of: *In-vitro* grown roots and callus; Leaves of field grown plant	Up to 83% activity from roots; 77% from leaves; 38% from callus	Shilpa et al., 2010
	Anti-diabetic activity	Methanolic root extract	Decreased blood glucose; Lowered levels of TC, LDL-C and VLDL-C; triglycerides, TBARS; Recovered serum insulin and tissue glutathione	Ahmad et al., 2012a
*O. basilicum*	Hepatotoxic activity	Different extracts from hairy roots, normal roots, aerial parts	Inhibition of membrane lipid peroxidation, cell necrosis, liver transaminases and MDA	Marzouk, 2009
	Anti-microbial activity	Rosmarinic acid	Growth inhibition, Morphological changes, cell wall deformation in: Fungi: *Phytophthora drechsleri*, *P. megasperma*, *P. parasitica*, *Aspergillus niger*, *Rhizoctonia solani*, *Fusarium oxysporum*, *Pythium aphanidermatum*, *P. ultimum*, *Verticillium dahliae*, *Alternaria solani*, *A. brassicae* Bacteria: *Xanthomonas. campestris* pv. vesicatoria, *Pseudomonas fluorescens* PFS rpos, ZC, *Erwinia carotovora*, *A. rhizogenes* (ATCC 15834), *P. aeruginosa* (strains PAO1, PA14)	Bais et al., 2002
		Root and leaves of potted plant (40% peat + 40% Coir + 20% tuff + fertilizers)	*S. enterica* counts in: Leaves: Decreased at 6 h; Undetected at 16–24 h; Sap: sigmoidal increase. Root: Unchanged	Bernstein et al., 2017
		Root and leaves of plant in pot hydroponic system	Reduction in viral plaque and bacterial colonies; Measurable plaque/colony after inoculation: MNV-1 from day-1 to day-3, *S. enterica* serovar Thompson FMFP 899 from day-3 to day-6	Li and Uyttendaele, 2018
*O. canum*, *O. sanctum*, *O. basilicum*	Anti-plasmodial activity	Extracts of root, leaf, stem, flower	Suppression of parasitaemia from *Plasmodium falciparum*; Root (IC_50_ 78.69–100 μg/ml) < Flower (IC_50_ 71.91–82.08 μg/ml) < Stem (IC_50_ 53.50–63.37 μg/ml) < Leaves (IC_50_ 35.58–53.50 μg/ml); *O. canum* < *O. sanctum* ≤ *O. basilicum*	Inbaneson et al., 2012
*O. campechianum*+ 8 plants of Yucatan	Radical scavenging activity (Spectrometer and/or spectrofluorometer analysis)	Ethanolic extracts of root, stem, leaf; traditional preparation	Inhibition activity against Vesperlysine and Pentosidine-like AGEs (IC_50_ > 1 mg/mL); DPPH free radical scavenging activity (EC_50_ 235–300 μg/ml)	Dzib-Guerra et al., 2016
*O. sanctum*, *O. kilimandscharicum*		Ethyl acetate extract of root	DPPH free radical scavenging activity: 85% of *O. kilimandsacharicum* and 80% of *O. sanctum*; Ferric reducing antioxidant power (FRAP) assay: 80.98 FSE of *O. kilimandsacharicum* and 80 FSE of *O. sanctum*; Total antioxidant capacity: 7.56 AsE of *O. kilimandsacharicum* and 11.31 AsE of *O. sanctum*;	Agarwal et al., 2017

*TC, total cholesterol; LDL-C, low-density lipoprotein cholesterol; VLDL-C, very low-density lipoprotein cholesterol; HDLC, high-density lipoprotein cholesterol; TBARS, thiobarbituric acid reactive substances; MDA, malondialdehyde; DPPH, 2,2-diphenyl-1-picrylhydrazyl; FSE, ferrous sulfate equivalents; AsE, ascorbic acid equivalent.

### Hepatoprotective Activity

**FIGURE 2:**
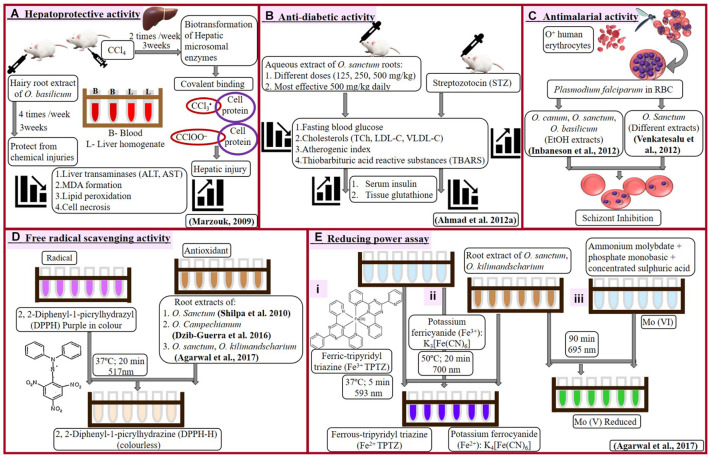
Hepatoprotective **(A)**, antidiabetic **(B)**, antimalarial **(C)**, and antioxidant (**D**-free radical scavenging and **E**-reducing power assay) activities of *Ocimum* sp.

Induced hepatotoxicity (using carbon tetrachloride, CCl_4_) in female albino rats resulted in enhanced lipid peroxidation, liver transaminases, and malondialdehyde (MDA), which ultimately leads to cell necrosis. Marzouk (2009) analyzed comparative hepatotoxic-inhibitory effect of extracts from aerial parts, normal roots, hairy roots, as well as purified six-triterpenes of *Ocimum basilicum*. Purified compounds, betulinic acid, and euscaphic aid (ursane derivative), and extract of hairy roots proved to be the best with hepatoprotective activity. Effect of CCl_4_ to induce hepatotoxicity and the counter-effect of hairy root extract has been presented in Figure 2A.

### Antidiabetic Activity

Ahmad et al. (2012a) examined blood glucose (fasting) and lipid profile in streptozotocin (STZ)-induced diabetic rats under influence of aqueous root extract of *O. sanctum* (Figure 2B). Even single dose of streptozotocin significantly elevated blood glucose, total cholesterol, low-density lipoprotein, very low-density lipoprotein along with reduction in levels of insulin and tissue glutathione (GSH). Administration of root extracts reduced elevated metabolites and recovered a level of insulin and GSH, which was more effective at a higher dose of 500 mg/kg. This biochemical profiling reveals the antidiabetic character of *O. sanctum* root extract.

### Antimalarial Activity

Antimalarial activity of *Ocimum* roots extracts against *Plasmodium falciparum* was analyzed by Inbaneson et al. (2012) and Venkatesalu et al. (2012). *P. falciparum* cultivated on O^+^ human erythrocytes was treated with different extracts of *Ocimum*. Inbaneson et al. (2012) studied ethanolic extracts of roots, stems, and leaves of *O. canum*, *O. sanctum*, *O. basilicum*; however, Venkatesalu et al. (2012) examined hexane, chloroform, ethyl acetate, acetone, and methanol extracts of *O. sanctum* roots along with different parts of six other plants (roots of *Adhatoda vasica*, pulp of *Carica papaya*, leaves of *Caesalpinia pulcherrima*, and *Erythroxylum monogynum*, as well as whole plants of *Lantana camara* and *Phyllanthus niruri*). Inhibition in parasitic growth was calculated as reduction in parasitemia by counting schizonts against 200 counts of an asexual parasite and expressed by inhibitory concentration (IC_50_) value.

Maximum extract yield (16.79%) and antiplasmodial activity were associated with leaves of *O. sanctum* (IC_50_ 35.58 μg/ml). Among *Ocimum* species, roots were remarked with lowest percentage yield of extracts (2.42–2.9%) and antimalarial activity (IC_50_ > 100 to 78.69 μg/ml), followed by flowers, stems, and leaves. However, roots of *O. basilicum* were most suitable to inhibit *P. falciparum* (IC_50_ 78.69 μg/ml) as compared with roots of other *Ocimum* species (Inbaneson et al., 2012). When compared with other plants, chloroform and methanolic root extract of *O. sanctum* (similar to methanolic extract of *Caesalpinia pulcherrima* and *Adhatoda vasica*) showed better antiplasmodial activity with minimum IC_50_ value of 11.47 and 14.04 μg/ml (Venkatesalu et al., 2012).

Treatment of *Ocimum* root extract prevented the erythrocytes injury and reduced spread of *P. falciparum* into fresh RBCs (Figure 2C). Such influence of *Ocimum* root extract can be very helpful in treating malaria, especially caused by drug-resistant *Plasmodium*.

### Antioxidant Activity

Shilpa et al. (2010) investigated DPPH (2, 2-Diphenyl-1-picrylhydrazyl) radical scavenging activity in ethyl acetate extract of leaves from field-grown as well as *in vitro* roots and callus of *O. sanctum*. Reaction to reduce purple-colored DPPH radical into colorless DPPH-H has been shown in Figure 2D. Increasing concentration (50–100 μg/ml) of *in vitro* (adventitious) root extract demonstrated increase in free radical scavenging activity from 73 to 83%. Callus was observed with minimum activity (38.6%), followed by leaves with 77% scavenging activity. *In vitro* roots reduced DPPH radical with 6% more efficiency as compared to *in vivo* leaves. This summarizes that extract of *in vitro* roots possesses maximum antioxidant activity as compared to *in vitro*-grown callus, leaves, or *in vivo* leaves.

Dzib-Guerra et al. (2016) studied many plants from Yucatecan traditional medicine system, including roots, leaves, and stems of *Ocimum campechianum* (Figure 2D). Aqueous traditional preparations, as well as different extracts of these plants, were analyzed for anti-advanced glycation end (AGE) product inhibition and DPPH radical scavenging activity. There was no significant anti-AGE activity observed with *O. campechianum* (IC_50_ > 1), which was maximum in *Cassia fistula* (IC_50_ 0.2–0.5). However, traditional preparation of *O. campechianum* exhibited better radical scavenging activity (EC_50_ 150) as compared to leaf, stem, and root extracts of the plant (EC_50_ 235 to > 300).

Agarwal et al. (2017) analyzed antioxidant potential of root extracts of *O. sanctum* and *O. kilimandsacharicum*, using different experiments (Figures 2D,E). Butanol, chloroform, ethyl acetate (EtAc), and hexane fractions of methanolic extract were used to test inhibition of DPPH activity and reduction of ferric to ferrous (using ferric tripyridyl triazine, Fe^3+^ TPTZ complex, as well as potassium ferricyanide). The ferric-reducing antioxidant power (FRAP) was calculated by comparing each *Ocimum* extract with the quantity of ferrous [ferrous sulfate equivalent required for the same change in optical density using FeSO_4_ as standard following the protocol of Luqman et al. (2012)]. Ethyl acetate fraction of *Ocimum* root extract holds maximum antioxidant potential followed by butanol, methanol, chloroform, aqueous, and hexane fraction. Root extract (EtAc fraction) of *O. kilimandsacharicum* possesses better DPPH scavenging potential (85 ± 2%) and ferric reduction (80.98 ± 3.50 ferrous sulfate equivalents, FSE) as compared with *O. sanctum* (80 ± 2%; 77.05 ± 3 FSE). Another test of iron reduction from potassium ferricyanide to potassium ferrocyanide expressed higher activity with EtAc fraction of *O. kilimandsacharicum* (1.64 ± 0.23) as compared with *O. sanctum* (2.14 ± 0.36) at 700 nm. Total antioxidant capacity was assessed as Molybdenum reduction from Mo (VI) to Mo (V) as ascorbic acid equivalent (AsE). Similarly, EtAc fraction of *O. kilimandsacharicum* and *O. sanctum* were detected with higher (7.56 ± 0.47 to 11.31 ± 1.02 μg AsE/mg, respectively) Mo reduction. Therefore, ethyl acetate extracts are best to prefer for antioxidant formulations using roots of *O. kilimandsacharicum* as compared with roots of *O. sanctum*. This finding suggests that root extract of *O. sanctum* is preferred, showing higher antioxidant potential over *O. kilimandsacharicum*.

### Effect of *Ocimum* on Physical Ability

**FIGURE 3:**
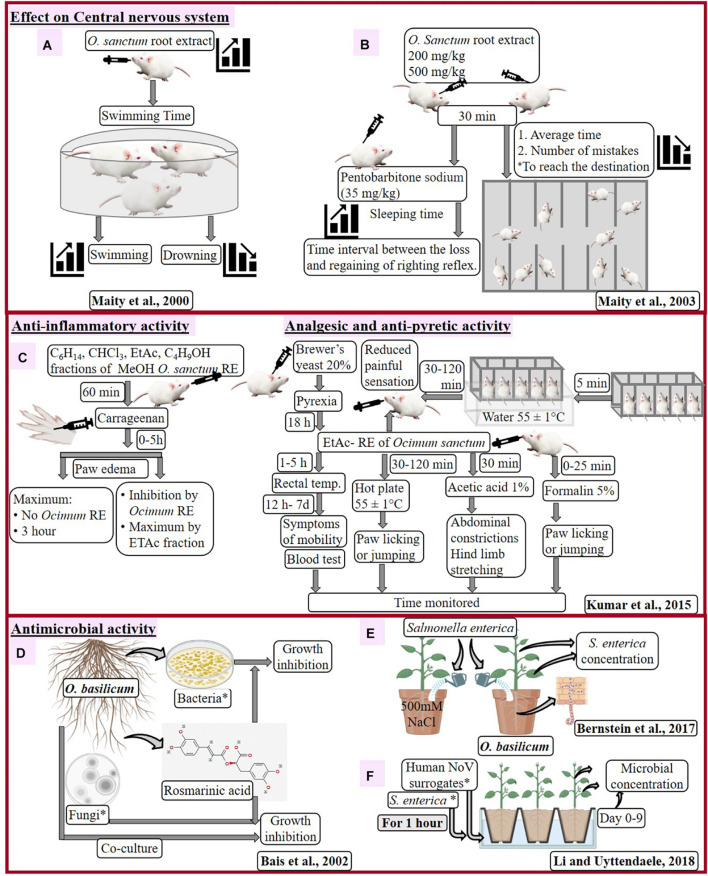
Effect of *Ocimum sanctum* root extract on central nervous system of mouse **(A,B)**, pharmacological activities of *O. sanctum* RE in the mouse model **(C)** and antimicrobial activity of *Ocimum basilicum* **(D–F)**. *C_6_H_14_, hexane; CHCl_3_, chloroform; EtAc, ethyl acetate; C_4_H_9_OH, butanol; RE, root extract; Bacteria: *Xanthomonas campestris*, *Pseudomonas fluorescens*, *P. aeruginosa* (strains PAO1 and PA14), *Erwinia carotovora*; Fungal isolates: *Alternaria brassicae*, *A. solani*, *Phytophthora megasperma*, *P*. *parasitica*, *P. drechsleri*, *P. ultimum*, *A. niger*, *Rhizoctonia solani*, *Fusarium oxysporum*, *Pythium aphanidermatum*, *Verticillium dahliae*; serovar Typhimurium SL 1344, serovar Thompson RM1987 and serovar Thompson FMFP 899; *S. enterica*: 3-strains serovar Typhimurium SL 1344, serovar Thompson RM1987 and serovar Thompson FMFP 899; human noroviruses (NoV) surrogates: Murine norovirus-1, i.e., MNV-1 and Tulane virus, i.e., TV.

Maity et al. (2000, 2003) analyzed effect of methanolic root extract from field-grown *O. sanctum* with swimming performance of adult male albino mice and pentobarbitone sodium-induced hypnosis in Swiss albino mice (Figures 3A,B). Dose-dependent decrease in mean swimming time of mice has been analyzed (Maity et al., 2000) with root extract treatment, along with dose-dependent decrease in number of errors and sleeping time (Maity et al., 2003). Effect of *O. sanctum* root extract on central nervous system was similar to antidepressant remedy and confirms adaptogenic activity of *O. sanctum* root.

Kumar et al. (2015) inspected effect of different fractions (hexane, chloroform, ethyl acetate, and butanol) of root methanolic extract of *O. sanctum* on Swiss albino mice for different disorders (Figure 3C). Treatment with root extracts (especially ethyl acetate, at 30 to 300 mg/kg) inhibited the paw edema (13–60%), showing anti-inflammatory activity, heat-induced painful sensation (31–51%), chemical-induced pain (22–31%), paw licking (22–51%), and number of writhing (24–53%), showing analgesic activity as well as reduction of temperature (29–38%), showing anti-pyretic activity in dose-dependent manner. Such response of *O. sanctum* root extract could defy several after effects observed due to nonsteroidal anti-inflammatory drugs, like diclofenac sodium, therefore healthier as anti-inflammatory, antipyretic, and analgesic agents.

### Effect of Roots of *O. basilicum* and Rosmarinic Acid on Microbes

Inhibition in different types of bacterial and fungal growth recommends rosmarinic acid (RA) with constitutive antimicrobial activity. Bais et al. (2002) enhanced accumulation of RA in hairy root cultures of *O. basilicum*, which positively correlated with treatment of fungal cell wall elicitors (CWE). However, in the testing compound, RA has negative effect on growth of microbes, as evidenced by the zone of inhibition (Figure 3D). Out of tested bacteria of rhizosphere, *Xanthomonas campestris*, *Pseudomonas fluorescens*, *P. aeruginosa* (strains PAO1 and PA14), and *Erwinia carotovora* displayed concentration-dependent increase in the zone of inhibition. Among these bacteria, *P. aeruginosa* (human pathogen) was highly sensitive to RA treatment. Increased inhibition was observed with higher titers and higher amount of RA in case of *Pythium ultimum* and *P. aphanidermatum*, respectively, while delayed inhibition was observed in *Aspergillus niger* and *Fusarium oxysporum* on increasing the concentration of RA. Microscopic examination reveals morphogenic alteration in fungi due to RA treatment like clumping of hyphae (*P. drechsleri*), rounded spores (in place of dumbbell shaped), damaged cell surface, damaged hyphae, broken intersepta (*A. niger*), prolific cell division, and damaged nucleoid along with condensed genetic material (*P. aeruginosa*). The antifungal mechanism of *O. basilicum* was briefly studied by co-culturing hairy roots with fungal hyphae. Root tips overcame fungal growth having droplets (exudates) with RA at the region of fungal contacts.

These findings suggest that metabolites like RA might get released in rhizosphere to combat microbial infection. The antimicrobial nature of *O. basilicum* roots and RA challenges infection from soil-born microbes.

### *In situ* Effect of *O. basilicum* on Human Pathogen

Contaminated soil and water with human pathogens help them to penetrate and contaminate plant tissues. Interaction of plants and pathogens influences morphological, molecular, and physiological changes in plants, as well as pathogens. Plant and pathogen interaction becomes more interesting and significant when it comes to widely used traditional medicinal plants. Bernstein et al. (2017) studied effects of *Salmonella enterica* serovar Newport (strain 96E01153C-TX) on *O. basilicum* (Figure 3E) as colony forming unit (CFU). Li and Uyttendaele (2018) examined the pathogenicity of *S. enterica* serovar Typhimurium (strain SL 1344) and *S. enterica* serovar Thompson (strains RM1987 and FMFP 899), along with two types of human noroviruses (NoV) surrogates, Murine norovirus-1 (MNV-1), and Tulane virus (TV) on *O. basilicum* (Figure 3F) as CFU and plague-forming unit (PFU). Effects of *O. basilicum* on these pathogens are discussed below:

Bernstein et al. (2017) inoculated soil, leaf (petiole), and leaf sap of 60-day-old *O. basilicum* (with and without 0–500-mM NaCl treatment) with 10^6^ CFU g/soil, 10^9^ CFU ml/l and 10^3^ CFU ml/l suspension of *S. enterica*, respectively. *Salmonella* concentration was determined in potting mixture, shoots, leaves, and leaf sap. Root penetration of *S. enterica* as well as its concentration in soil, leaves, and leaf sap was not affected by salt treatment. *Salmonella* was undetected in aerial parts of the plant when inoculated in soil. *S. enterica* counts in leaves reduced by 2.9 × 10^3^ CFU/g/day within 6 h and becomes undetected after 16–24 h. However, sigmoidal increase in growth of *S. enterica* was observed in leaf sap of plants. Concentration of *S. enterica* and total aerobic bacteria were decreased (1–1.5 log CFU/g) from 24 h to 5 days (Bernstein et al., 2017). Although salt stress induces lots of changes (root branching, root mass, stomatal conductance, and transpiration rate) in plants, it did not affect interaction of pathogen with *Ocimum*. Salinity neither affected survival of *S. enterica* nor its integration into roots of *O. basilicum*. Metabolic composition, as well as active innate immunity of intact leaves, attributed to the growth inhibition of *S. enterica*, as compared with leaf sap.

Li and Uyttendaele (2018) prepared 8.46 log-PFU/ml and 8.60 log-CFU/ml solutions of NoVs and *S. enterica* strains, respectively, to infect leaves of 4-week-old and roots and 6-week-old *O. basilicum*. Pathogens were quantified using titration by plaque assay for virus as well as pre- and post-enrichment quantification for bacteria. Consistent reduction of >5.5-log, >3.3-log, and 3 to 4-log was observed with MNV-1, TV, and *S. enterica* strains, respectively, in leaves after 3 days of inoculation through leaves. *S. enterica* Thompson FMFP 899 can be identified as most infectious due to better survival with 5.5 to a 6.7-log CFU/sample leaf from 6 to 9 days of leaf infection. Internalization of pathogens *via* roots toward leaves was tested for MNV-1 and *S. enterica* serovar Thompson FMFP 899 after 1 h, followed by 1, 3, 6, and 9 days. MNV-1 was reduced from 540, 580, 400 PFU/g tissues (at Day 1) to 280, 80, < 4 PFU/g tissues (at Day 3); after that, virus was unable to detect. Only one in three colonies of *S. enterica* serovar Thompson FMFP 899 was detected after 3–6 days with *S. enterica* serovar Thompson FMFP 899.

Human noroviruses and *S. enterica* are most infectious pathogens of food industry with maximum transmission and internalization into plant tissues due to soil and water contamination. Time-dependent reduction of these pathogens was observed after infection *via* leaves and roots due to antimicrobial nature of the plant. These pathogens negatively affect the food crops, including lettuce, green onion, kale, mustard, and spinach. These pathogens spread faster due to highly contaminating nature; however, antimicrobial nature of *Ocimum* prevents their growth.

## Effect of Rhizosphere on *Ocimum* Plants

Rhizosphere affects growth and health of overall plants. Therefore, biotic factors like bacteria and nematodes, as well as abiotic ones, such as salt, greatly influence the overall significance of the medicinal plants (Table 2).

**TABLE 2:** Effect of microorganisms (nematode, deaminase bacteria, and symbiotic fungi) and different abiotic stresses (salt heavy metal and radioactive metal) on roots and the whole plant of *Ocimum* sp.

Plant	Treatments	Growth condition	Findings	References
*O. basilicum*	*Meloidogyne incognita*, *Belonolaimus longicaudatus*, *Dolichodorus heterocephalus*, *Paratrichodorus christiei*, *Pratylenchus scribneri*, *Hoplolaimus galeatus*,	Myakka fine sand (92% sand, 6% silt, 2% clay)	Heavy galling, necrosis; stubby roots, swollen tips, darkened lesions; length of secondary roots; dead roots, brown lesions, decay	Rhoades, 1988
	Cr(III) as CrCl_3_.6H_2_O (0, 2, 4, 6, 8 mg/L)	Hoagland media	**TEM:** Indistinct plasmalemma, cell organelles within distorted root and leaf cells; Dense, normal and seldom granular deposits in root cells **Heavy metals (in roots and shoots):** Higher: Cr; Lower: Cu, Zn, Co, Ni, Mo	Bishehkolaei et al., 2011
	Thiuram (fungicide; 6 mg/kg of soil)	Mineral soil-A; Organic soil-B;	**Enhanced:** Plat growth, Physiological activities, heavy metal accumulation	Adamczyk-Szabela et al., 2017
	NaCl (30 mM)	40% peat + 40% Coir + 20% tuff + fertilizers	**Reduced:** Plant growth, Level of K and Ca, Relative water content (6%), CO_2_ fixation (29%), stomatal conductance (26%) **Enhanced:** Na (4-fold), Cl (9-fold), osmotic potential (30%), antioxidant activity (30%), essential oil (26%)	Bernstein et al., 2017
	*Rhizophagus irregularis* and *Serendipita indica*; Pb(NO_3_)_2_.7H_2_O; CuSO_4_.5H_2_O	50% peat + 50% sand	**Reduced:** Eugenol **Enhanced:** fresh and dry weight of roots and shoots; phosphorus; linalool; eucalyptol; Methyl chavicol; **Conc. of Pb and Cu:** decreased in shoots, increased in roots	Sabra et al., 2018
	KCl, K_2_SO_4_ and Khazra-K-nano-chelate in soil for K (0, 100, 200 mg/kg)	Silty clay loam calcareous soil	**Enhanced:** Conc. In roots: Zn (up to 28%); Conc. in shoots: Cu (up to 30%), Mn (14%); Translocation: Zn, Cu **Reduced:** Conc. in roots: Cu (up to 42%), Mn (up to 20%)	Zahedifar et al., 2019
	Cd(NO_3_)_2_ in soil for Cd (0, 40 mg/kg) + KCl, K_2_SO_4_ and Khazra-K-nano-chelate in soil for K (0, 100, 200 mg/kg)		**Enhanced:** Translocation: Cd (up to 86%), Fe (NA); Conc. in shoots: K (11%), Cd (up to 89%), Fe (up to 39%); Shoot uptake: K (up to 14%); **Reduced:** Conc. in shoots: Zn (up to 18%), Cu (up to 18%); Mn (up to 28%); Shoot uptake: Cu (up to 31%); Mn (up to 41%) Translocation: Cd and Mn.	
*O. tenuiflorum* (*O. sanctum*)	Cr(VI) as K_2_Cr_2_O_7_⋅7H_2_O (0.0, 10.0, 20.0, 50.0, 100.0 μM)	Acid washed sand (0.01 M HCl); Hoagland media	**Enhanced:** NR activity, protein, Nitrate, Proline, MDA, potassium leakage, SOD, GPX, catalase **Reduced:** Plant growth, chlorophyll, carotenoids, ascorbic acid, cysteine, NP-SH, APX	Rai et al., 2004
	Ten- ACC deaminase bacteria isolated from rhizosphere; Especially four: *Achromobacter xylosoxidans* (Fd2), *Serratia ureilytica* (Bac5), *Herbaspirillum seropedicae* (Oci9) and *Ochrobactrum rhizosphaerae* (Oci13)	Waterlogging	**Enhanced:** shoot fresh weight, shoot length, number of nodes, no. of leaves, Chlorophyll, foliar-P, N-uptake, K^+^ **Recovered:** Root weight **Essential oil:** Germacrene-D, β-elemine, β-caryophyllene **Reduced:** Proline, MDA, ACC level	Barnawal et al., 2012
	Natural habitat	Naturally grown pasture and forest region	**Maximum mean activity conc.** (Becquerel/kg DW): **In leaves:**^210^Po: 63.7, ^210^Pb: 406; **In soil:**^210^Po: 17.1, ^210^Pb: 58 **Transfer factor:**^210^Po: 3.7, ^210^Pb: 7.0	Chandrashekara and Somashekarappa, 2016

* MNV-1, murine norovirus-1; TV, Tulane virus.

### Growth-Promoting Bacteria in Rhizosphere of *O. sanctum*

Rhizosphere of *Ocimum sanctum* was analyzed by Barnawal et al. (2012) under waterlogged condition to isolate bacteria containing 1-aminocyclopropane-1-carboxylic acid (ACC) deaminase. Among isolated 10 bacteria, four strains were verified for growth-promoting activity for waterlogged plants and identified as *Achromobacter xylosoxidans* (Fd2), *Serratia ureilytica* (Bac5), *Herbaspirillum seropedicae* (Oci9), and *Ochrobactrum rhizosphaerae* (Oci13) through rDNA sequencing. Barnawal et al. (2012) studied *O. sanctum* plants (10 weeks old) after treating with isolated 10-bacterial strains for 4 weeks and flooded them for the next 15 days.

Strain Fd2 was observed with maximum growth-promoting effect under waterlogging, with maximum increase in shoot FW (46.5%), shoot length (76.3%), number of nodes (72%), number of leaves (41.9%), and maximum recovered root weight (37%). Non-waterlogged *O. sanctum* plants exhibited maximum shoot FW (59.8%) and minimum reduction in root weight (16%) with Oci13 strain, while maximum shoot length (22%), number of leaves (26.8%), and number of nodes (39.4%) with Bac5 strain. Germacrene-D, β-elemine, and β-caryophyllene were observed as major components of essential oil, with no significant changes due to waterlogging or ACC-deaminase-containing rhizobacteria. Enhanced chlorophyll content in *O. sanctum* was caused by waterlogging reduction and in the presence of rhizobacteria. Inoculation with Fd2 resulted in maximum chlorophyll content (49.5%) in waterlogging. Inoculation with ACC-deaminase bacteria reverted the effects of waterlogging by maximizing reduction in elevation of proline (18%), MDA [22%, by strain Fd2 and the ACC level (62%, by Bac5 strain)]. However, strains Oci13, Oci9, and Fd2 were observed with maximized foliar-P (22.5%), N-uptake (13.6%), and K^+^ content, respectively (Barnawal et al., 2012).

Study of Barnawal et al. (2012) emphasized significance of microbes like ACC-deaminase-containing rhizobacteria in growth of *O. sanctum* and also to overcome the effects of stresses like waterlogging.

### Effect of Different Nematodes on Growth of *O. basilicum*

Rhoades (1988) analyzed soil and root of unhealthy *O. basilicum*. Roots of these plants were damaged due to the presence of few nematodes, *Meloidogyne incognita*, *Belonolaimus longicaudatus*, *Dolichodorus heterocephalus*, *Pratylenchus scribneri*, *Hoplolaimus galeatus*, and *Paratrichodorus christiei*. To confirm pathogenicity of these nematodes, Rhoades (1988) poured suspension of eggs, juveniles, and adults of different nematodes around rooted cutting (10–12-m tall) of the plant. Eggs of *M. incognita* (5,000 or 15,000 per pot), as well as juveniles and adults of *Belonolaimus longicaudatus*, *Dolichodorus heterocephalus*, *Pratylenchus scribneri* (l,000 or 2,000 per pot), *Hoplolaimus galeatus* (7,500 or 15,000 per pot), and *Paratrichodorus christiei* (150 per pot) were used to make suspension. Rhoades (1988) evaluated soil, plant growth, and yield, as well as root injury till 10 months. Plant growth and yield were unaffected till 5 months of all nematode inoculation. *D. heterocephalus* and *H. galeatus* had no significant effect on growth of *O. basilicum*, even after 5 months; however, other nematodes significantly reduced growth and yield from 5 to 10 months. Different nematodes injured roots in different ways. *M. incognita* caused heavy galling with necrosis, *B. longicaudatus* produced stubby roots with swollen tips and darkened lesions, *P. christiei* reduced length of secondary roots, while *P. scribneri* almost killed roots of *O. basilicum*.

Several nematodes can be pathogenic to *Ocimum* and compromise the production. Therefore, prior to cultivation, soil must be examined for pathogenic organisms. Abd-Elgawad (2021) and Renčo et al. (2021) confirmed the wide availability and pathogenic effect of these nematodes along with other species of nematodes.

### Salt Stress to *O. basilicum*

Bernstein et al. (2017) briefly studied the effect of saline treatment (30 mM of NaCl) on growth, physiology, as well as essential oil and metal accumulation in seedlings of *O. basilicum* (30 days old), for 30 days. Although salinity reduced shoots, roots, along with areas of individual leaves, shoots were observed with utmost reduction. Exposure of NaCl-enhanced Na (4-fold) and Cl (9-fold) with reduction in the levels of K and Ca. Salinity-induced reduction in relative water content (6%), CO_2_ fixation (29%), and stomatal conductance (26%) was observed along with increase in osmotic potential (30%), antioxidant activity (30%), and essential oil (26%) content.

Bernstein et al. (2017) highlighted the negative impact of increased salinity in soil on *O. basilicum*, which significantly reduced the growth, physiology, and metabolic contents of the plant.

### Metal Uptake With Different Soil Compositions and Fungicide Treatments

Roots are primarily considered as the transport system to provide water and nutrients (present in soil) to aerial parts of plants. This way, it becomes essential to understand the performance of the root system in the current scenario of soil pollution for translocation of different metals. Adamczyk-Szabela et al. (2017) examined growth and physiological activities of *O. basilicum* (1 month old) germinated on two different soil types with and without thiuram (fungicide) treatment for 14, 28, and 42 days (Table 2). Soil-A from uncultivated farmland (for 2 years) and soil-B from organic rich commercial land were confirmed with acidic nature and no heavy metal contamination. Time-dependent increase in all physiological activities was observed in plants of both soil-A and soil-B, which further enhanced after fungicide treatment, except for intracellular CO_2_.

Roots of *Ocimum basilicum* (grown in both soils) were observed with higher content of Co, as well as lower content of Cd and Zn at 14th day, which alters till 42nd day of fungicide treatment. The accumulation patterns of Pb, Ni, and Mn for soil-A and Cu for soil-B were similar to Co. Similarly, Cu and Pb showed a similar pattern of Cd, with soil-A and soil-B, respectively. Mn content in shoots of *O. basilicum* gradually increased in soil-A, while, in soil B, increased at 42nd day after gradual decrease till 28th day of thiuram treatment. Thiuram treatment had no effect on Mn translocation factor (TF, metal distribution), which was highest with both soils, as well as Zn and Cd uptake (Transfer coefficients, TC), which was maximum with soil-A and soil-B, respectively. However, TCs for other metals vary with treatment. With plants of soil-A, TF for Mn and Zn was increased at 14th day, and then gradually decreased. Plants grown in soil-B were observed with higher TF value for Mn, Zn, Pb, and Ni on 42nd day of thiuram treatment.

Thiuram has low water solubility and rather adsorbs to soil particles. It degrades easily in organic soil (acidic). Thiuram enhanced metal uptake in soil-A (for Co, Pb, Ni, and Mn) as compared with soil-B (Co and Cu). Degradation of thiuram due to existing humic acids and lesser accessibility of metals due to stable metal complexes in soil B is the reason behind lower heavy-metal uptake in soil-B. Therefore, it is important to consider the nature of soil and plantation of *Ocimum* and requirement of fungicide.

### Metal Uptake With Different Forms of Chromium Contamination

Heavy utilization of chromium is well-known in catalyst application, electroplating, leather tanning, pigment manufacturing, metal finishing, and steel production. Gigantic consumption of carcinogenic chromium becomes the cause of soil and water pollution. Due to therapeutic nature of *Ocimum*, Cr exposure to *O. tenuiflorum* as Cr(VI) using K_2_Cr_2_O_7_⋅7H_2_O (0 to 100 μM), as well as to *O. basilicum* as Cr (III) using CrCl_3_.6H_2_O (0–8 mg/l), was examined by Rai et al. (2004) and Bishehkolaei et al. (2011), respectively (Table 2).

Chromium concentration increased in plant tissues with increase in Cr concentration and duration of exposure. Roots of *O. tenuiflorum* accumulated more Cr as compared to leaves of same. Highest accumulation of Cr (419.50 μg g^–1^ dry weight, DW) was observed in roots of *O. tenuiflorum* at 72 h of 100-μM Cr treatment. Chromium uptake reduced plant growth, biomass, total chlorophyll, chlorophyll a, chlorophyll b, and carotenoids in dose- and time-dependent manner. Similar to plant growth, maximum reduction in nitrate reductase activity (64%), protein content (77.45%), and accumulation of nitrate (3.59-fold) was recorded with 100-μM Cr exposure for 72 h in leaves. Same conditions inhibited ascorbic acid (52.83%), cysteine (77.33%), nonprotein thiol (NP-SH, 60.87%), and activity of APX (76.22%). However, proline content (574.94 μmol g/FW), MDA content (5-fold), potassium leakage (which leads to cell membrane disruption), as well as activities of SOD (3.29-fold), GPX (5.56-fold), and catalase (1.63-fold) were enhanced with increasing dose and time. TLC of leaf tissues confirmed enhanced eugenol accumulation due to Cr exposure. However, maximum increase in eugenol content (24.61%) was observed with treatment of 20-μM Cr for 72 h. Based on physiological and metabolic changes, *O. tenuiflorum* can tolerate chromium-induced stress (Rai et al., 2004).

Root cortical and leaf mesophyll cells were analyzed through TEM, which reveals indistinct plasmalemma and cell organelles within distorted cells when treated with higher concentrations (4–8 mg/l) of Cr (III). Root cortical cells of Cr (III)-treated plants exhibited dense, normal, and seldom granular deposits along the periplasmic zone of cell wall, the innermost layer of cell wall, and cytoplasm, respectively. Granular deposits of root cortical cells consist of Cr as the dominant element along with Cu, Zn, Co, Ni, and Mo. Increasing concentration of (≥4 mg/l) Cr (III) treatment resulted in decreased amount of other heavy metals in roots as well as shoots. This could be due to either Cr-inhibited activity of H^+^- ATPase associated with plasma membrane, or competitive interaction with plasma membrane. However, accumulation of Cr was higher in roots (up to 1130.9 ± 51.5-mg/g DW) as compared to shoots (up to 57.2 ± 2.7-mg/g DW) of *O. basilicum*, with maximum concentration of Cr (Bishehkolaei et al., 2011).

*Ocimum tenuiflorum* exhibited reduced growth and chlorophyll with enhanced eugenol and antioxidant enzymes as protective measures to stress. Both, *O. tenuiflorum* and *O. basilicum* accumulated lesser Cr in shoots as compared to roots. Cr (IV) gets reduced to Cr(III), which later confiscated in the vacuole of root cells in order to prevent Cr translocation in aerial parts of the plant. This is the natural defense mechanism to prevent Cr toxicity in *Ocimum* plants and is the reason behind the adaptive behavior of *O. tenuiflorum* and *O. basilicum* during chromium exposure (Rai et al., 2004; Bishehkolaei et al., 2011).

### Metal Uptake With Natural Radioactive Elements

Chandrashekara and Somashekarappa (2016) analyzed activities of natural radionuclides ^210^Po and ^210^Pb in leaves of *O. sanctum* (natural habitat) and associated soil samples, along with other 11 medicinal plants and 11 soil samples (Table 2). Mean activity concentration of radionuclides varied from 11.2 to 42.9 Becquerel/kg DW in soil and 3.3 to 63.7 Becquerel/kg DW in tested plants for ^210^Po, as well as 36.1 to 124 Becquerel/kg DW in soil and 12 to 406 Becquerel/kg DW in tested plants for ^210^Pb. Leaves of *O. sanctum* were observed with maximum mean activity concentration of both radioactive elements ^210^Po (63.7 Becquerel/kg DW) and ^210^Pb (406 Becquerel/kg DW) among 12 selected medicinal plants. However, soil samples related to *O. sanctum* were observed with medium range mean activity concentration ^210^Po (17.1 Becquerel/kg DW) and ^210^Pb (58 Becquerel/kg DW). Higher concentration of elements in leaves suggested more translocation from roots, which was proved with observed maximum transfer factor (TF) of *O. sanctum* for ^210^Po (3.7) and ^210^Pb (7.0) as compared with other 11 medicinal plants. Chandrashekara and Somashekarappa (2016) calculated these natural radionuclides to understand the safety of medicinal plants based on their radiological behavior. Chandrashekara and Somashekarappa (2016) assumed the role of leave morphology, surface area, shedding time, age of leaf, along with height of the plant in the uptake of ^210^Po and ^210^Pb. The documentation of radionuclides uptake and accumulation in medicinal plants like *Ocimum* are necessary steps before consuming them in the drug and food industry.

### Metal Uptake With Pb and Cu Contamination Affected by Endophytic Fungi

Sabra et al. (2018) treated potting mixture of *O. basilicum* (4 weeks old) with *Rhizophagus irregularis* (AM) and/or *Serendipita indica* (beneficial endophyte) for 5 weeks, as well as with 400 or 200 mg/l of Pb [as Pb(NO_3_)_2_.7H_2_O] and/or Cu (as CuSO_4_.5H_2_O) for 10 days. Maximum mycorrhization of *R. irregularis* (63%) in roots of *O. basilicum* when inoculated with both fungi, however, reduced to 46% in the presence of both heavy metals. Highest increase in fresh and dry weight of roots and shoots of plants was testified with *R. irregularis* alone, followed by treatment of *S. indica* and both fungi. Treatment of *S. indica* positively influenced shoot and root dry weight with and without contaminating Pb and Cu, respectively. P uptake in shoots by plants was highly promoted by *R. irregularis* with Pb and/or Cu contamination; however, *S. indica* promoted P uptake with and without Pb treatment only. Maximum reduction in shoot Pb content (48%) was observed with treatment of both fungi, followed by *R. irregularis* and *S. indica* in Pb contamination. However, Cu content in shoots decreased with treatment of *S. indica* and both fungi on Cu- and/or Pb-contaminated soil. Pb and Cu contents in roots were enhanced in roots grown on soil treated with both fungi and metal treatment. Single-metal treatment had no significant effect on root metal accumulation. Effect of fungal and metal treatments on metabolites in shoots of *O. basilicum* was also analyzed by them. *R. irregularis* enhanced the level of two phenylpropenes, linalool (1–7-mg/g DW) and eucalyptol (from 0.1-to-0.8-mg/g DW) even in uncontaminated or single-metal (Pb or Cu)-contaminated condition. Eugenol (terpene) content was reduced from 2.2 to 0.5-mg/g DW, only with *S. indica* colonization along with Pb treatment. Methyl chavicol (terpene) was enhanced from 5- to 7.2-μg/g DW by mycorrhization of roots in only uncontaminated soil.

Sabra et al. (2018) discussed the involvement of beneficial fungi to promote growth and metabolic composition of *O. basilicum* and overcome aftereffects of heavy metal stress (Table 2). Variations in the accumulation pattern of phenylpropenes and terpenes were observed due to unrelated biosynthetic pathways. Such growth-promoting microbes become noteworthy to endorse plant growth in tough environmental circumstances.

### Metal Uptake Affected by Different K-Fertilizers and Cd Treatments

Zahedifar et al. (2019) germinated seeds of *O. basilicum* in soil treated with Cd as Cd(NO_3_)_2_ (0- and 40-mg/kg soil), along with KCl, K_2_SO_4_, and Khazra-K-nano-chelate ( 0-, 100-, 200-mg/kg soil), for 10 weeks. Cd treatment increased K concentration in shoots of *O. basilicum* by about 11% in all K-treatments, while the shoot uptake was decreased by 12, 14, and 8% with KCl, K_2_SO_4_, and K-nano-chelate. The use of K in the form of K-nano-chelate reduced Cd accumulation. The potassium supplement (without Cd) enhanced Zn (up to 28%) in roots, Cu (up to 30%), and Mn (14%) in shoots as well as reduced Cu (up to 42%) and Mn (up to 20%) in roots of *O. basilicum*. Lesser stability of CdCl_2_ (as compared with CdSO_4_) enhanced the phyto-availability of Cd, hence accumulation of Cd in shoots with 40-mg Cd and 100-mg KCl. Enhanced translocation (86, 82, and 76%) of Cd from roots to shoots, as well as Cd concentration (89, 86, and 81%) in shoots, was observed with 40 mg of Cd along with different K-treatments. Cd treatment (with the K supplement) reduced shoot concentrations of Zn (up to 18%), Cu (up to 18%), Mn (up to 28%), shoot uptake of Cu (up to 31%), and Mn (up to 41%) of *O. basilicum*. Increasing concentration of Cd possesses inhibitory effect on translocation of Cd and Mn, which was also reduced due to K supplementation. Treatment of Cd enhanced shoot Fe (up to 39%) and Fe transfer from roots to shoots, which was further accompanied by the K supplement. Translocation of Zn and Cu seemed to be unaffected by Cd treatment and increased by the K supplement. Transfer of Cd from roots to shoots was observed maximum with K in the form of nano-chelate. Table 2 explains the effect of different K-fertilizers on heavy metal uptake in roots and shoots of *O. basilicum* in Cd-contaminated soil. The result signifies K-nano-chelate as a preferable source of potassium fertilizer for the growth of *O. basilicum*.

## Root Uptake of Nanoparticles and Their Effect on the Whole Plant

Study and involvement of nanoparticles are increasing every day. Nanoparticles are being used in several industries, including medicine, paint, plastic, and agriculture. These nanoparticles can affect plants through direct use in agriculture or indirect as a waste or a contaminant. The effect of nanoparticles in *O. basilicum* has been extensively studied by (Tan et al., 2017, 2018a, b) using nanoparticles of Ti and Cu.

### TiO_2_ Nanoparticles as Pollutants

**FIGURE 4:**
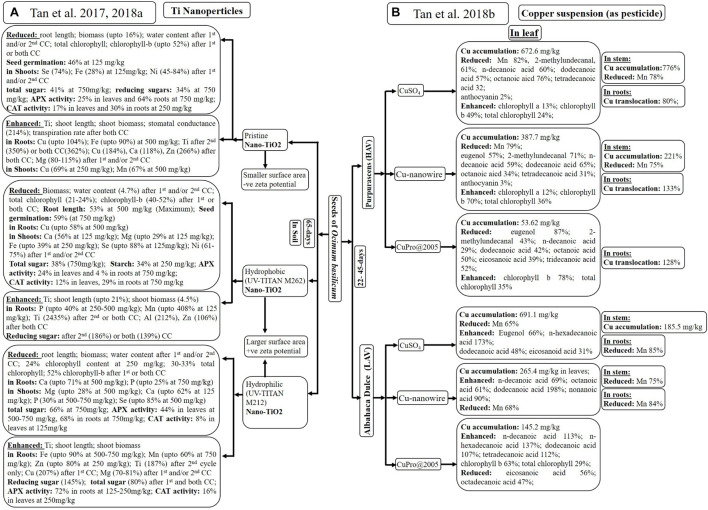
Analysis of plant growth as well as accumulation of elements and metabolites after introduction of: Ti-nanoparticles **(A)** and Cu-suspensions **(B)** through root system of *Ocimum basilicum.* *CC: cultivation cycle; HAV: High anthocyanin variety; LAV: Low anthocyanin variety.

Tan et al. (2017, 2018a) studied plants germinated on soil treated with 0–750 mg/kg of three different types of nano-TiO_2_ (unmodified, hydrophobic, and hydrophilic) for first (Tan et al., 2017), second, or both cultivation cycles (Tan et al., 2018a). Subsequently, growth parameters, physiological activities, antioxidant enzyme activities, and nutrient/metal distribution were examined. Details of experiments have been mentioned in Figure 4A.

Tan et al. (2017, 2018a) observed maximum Ti accumulation with hydrophobic nanoparticles, although all nanoparticles enhanced Ti content in a dose-dependent manner. There was no significant difference in Ti translocation from roots to shoots using any of nano-TiO_2_ (Tan et al., 2017). Tan et al. (2017, 2018a) experienced variability in macro- and micronutrient content with different types of nano-TiO_2_ at different concentrations. Variability among few nutrients in leaves and shoots has been given in Figure 4A.

Only hydrophobic and unmodified nano-TiO_2_ inhibited seed germination (up to 59%), root length (up to 53%), biomass (up to 16%), and water content (up to 16%) of *O. basilicum* (Tan et al., 2017). However, higher concentration of nanoparticles appeared to have no effect on the germination rate, root length, the photosynthetic rate, and chlorophyll-a in any cultivation cycle (Tan et al., 2018a). Tan et al. (2018a) observed maximum increase in shoot length (up to 21%) and shoot biomass (4.5%) after first and/or second cycle of treatment with hydrophobic nanoparticles, followed by uncoated and hydrophilic nano-TiO_2_. Nanoparticles treatment during first and/or both cycles reduced water content (up to 4.7%) total chlorophyll (up to 33%) and chlorophyll-b (up to 52%). Tan et al. (2017, 2018a) mostly experienced reduction in total sugar, reducing sugars and starch after nanoparticles treatment. Only treatment with hydrophobic nanoparticles for second (186%) or both cycles (139%) enhanced reducing sugar in *O. basilicum*. All nano-TiO_2_ significantly reduced APX activity in leaves (up to 44%) and roots (up to 29%) of *O. basilicum* at 500–750 mg/kg of nano-TiO_2_. However, only hydrophilic nanoparticles first enhanced APX activity (72%) in roots at lower concentration (125–250 mg/kg) due to interference of the -OH group.

Being used in several industries, nano-TiO_2_ gets released in water and soil, which ultimately affects the plants. Tan et al. (2017, 2018a) presumed that, by changing the properties of nano-TiO_2_, their uptake and effect on plants can be reduced. All three types of nanoparticles affected the plant in different ways, which was continued for the next cultivation cycle. Pristine nano-TiO_2_ enhanced gas exchange; hydrophobic nano-TiO_2_ increased shoot length and biomass, reducing sugar with reduced chlorophyll contents in one or both cultivation cycles. Hydrophilic nano-TiO_2_ reduced total sugar in first cultivation cycle, which was improved in the next cycle. Therefore, selection of nanoparticles totally depends on the desired morphological or physiological character.

### Cu-Nanowire- as Pesticide

Tan et al. (2018b) studied effect of Cu suspensions [CuSO_4_, Cu(OH)_2_ nanowires and CuPro@2005, as pesticides] on high and low anthocyanin varieties of *O. basilicum* (22 days old) through spray every 3rd day (till 4.8-mg Cu/pot). The differences in physiological activities, metal as well as metabolic accumulation of dark opal, “Purpurascens” (high-anthocyanin variety, HAV) and dulce, “Albahaca Dulce” (low anthocyanin variety, LAV) varieties (45 days old), are mentioned in Figure 4B.

Ionization of Cu^2+^ with CuSO_4_ suspension results in higher accumulation of Cu in leaves (691.1 mg/kg) and stems (185.5 mg/kg) of LAV, followed by leaves (672.6 mg/kg) and stems (776%) of HAV plants. Only HAV variety demonstrated higher Cu translocation from shoots to roots, using CuSO_4_ (80%), Cu(OH)_2_ nanowires (133%), and CuPro (128%) due to more dissolution of Cu in acidic nature of HAV leaves. Smaller size of Cu(OH)_2_ nanowires promoted accumulation by facilitating stomatal entry of nanoparticles as compared to CuPro@2005. Only Mn content reduced in leaves (65–82%), stems (up to 78%), and roots (up to 84%) of both varieties after exposure of CuSO_4_ and Cu(OH)_2_ nanowires. Cu-induced alteration in Mn has been reported in several other plants.

Tan et al. (2018b) observed elevated levels of most of the important metabolites, including eugenol (66%), in LAV variety. Variety HAV exhibited reduction in 2-methylundecanal (71, 43, and 61%) and eugenol (57 and 87%) when treated with Cu(OH)_2_ nanowires, CuPro@2005, and CuSO_4_, respectively. Handling of CuSO_4_, Cu(OH)_2_ nanowires, and CuPro@2005 with HAV variety dropped the concentration of n-decanoic acid (60, 59, and 29%), dodecanoic acid (57, 65, and 42%), and octanoic acid (76, 34, and 50%), respectively. However, the same Cu suspensions enhanced dodecanoic acid (48, 198, and 107%), n-decanoic acid (0, 69, and 113%), octanoic acid (20, 61, and 5%), and nonanoic acid (55, 90, and 10%) in LAV variety. Saturated fatty acids like eicosanoic acid (56%) and octadecanoic acid (47%) were decreased with CuPro@2005 treatment due to its negative effect on LAV variety. Enhanced chlorophyll content was observed with only CuPro@2005 in LAV and with all three treatments in HAV.

Anthocyanin content influenced Cu uptake along with physiological and metabolic changes in presence of Cu suspensions. Acidic nature of HAV, as well as higher accumulation of Cu, promoted more chloroplast development and reduction of essential oil as well as fatty acids, as compared to LAV. Application of Cu as nanowire has least Cu accumulation and translocation along with enhanced fatty and essential oil in LAV as compared to HAV. Therefore, Cu nanowire can be used as potential pesticide for *O. basilicum*.

## Adventitious Root Culture

The most vital part of the plant – roots – exists underground and helps in healthy growth and stability of the plant. Economic benefits increase the significance and the demand of *Ocimum* roots, which get affected by rhizosphere. Therefore, *in vitro* studies on roots of medicinal plants like *O. sanctum* seek attention of researchers.

**FIGURE 5:**
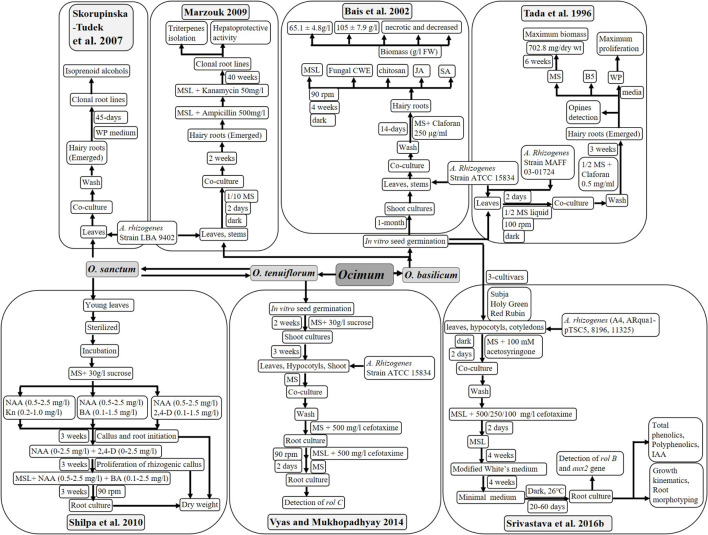
Hairy root and *in vitro* root cultures of *Ocimum basilicum* and *Ocimum sanctum.*

Till date, only Shilpa et al. (2010) mentioned the development of adventitious *in vitro* root cultures (Figure 5). Sterilized leaves were inoculated on MS media supplemented with different concentrations of NAA, in combination with BA, kinetin or 2, 4-dichlorophenoxyacetic acid. Root proliferation was achieved in obtained rhizogenic calli on MS media containing auxins only. MS liquid supplemented with different concentrations and combinations of NAA and BA was used for the further subculture of roots. Maximum callus induction was observed during the 1st week of culture, caused by NAA (2 mg/l) in combination with Kn (0.2 mg/l) or BA (1.3 mg/l), as well as NAA (0.5 mg/l) with 2,4-D (1.5 mg/l). However, only NAA with Kn showed root induction during the 2nd week of culture. Further root growth was achieved when induced roots were transferred to MS media containing NAA (0.5 mg/l) with 2,4-D (1.5 mg/l). No roots formation was observed with MS media with or without 2,4-D in the absence of NAA. Maximum root biomass of 1,460 ± 0.8 mg/l (dry weight) was accomplished in the liquid MS medium supplemented with 4 mg/l of NAA and 1.3 mg/l of BA. Root induction reduced with the reducing concentration of NAA, and callus induction increased with increasing concentration of BA. Therefore, the medium with the high auxin to cytokinin ratio is best for *in vitro* root culture.

*In vitro* root cultures present a better way to accumulate secondary metabolites with medicinal significance. In case of *O. sanctum*, NAA, in combination with BA, is required for rapid growth of adventitious roots from leaf explants and achieving maximum biomass.

## Hairy Root Culture*: Agrobacterium rhizogenes*-Mediated Transformation

Similar to *in vitro* root culture, hairy roots are the best mode to enhance biomass and accumulate metabolites specific to root tissues using bacterial infection. Gram negative bacteria *Agrobacterium rhizogenes* induce the hairy root production with the help of their rol-genes, which integrate into plant DNA after infection. These hairy roots can be cultured *in vitro* at a large scale using bioreactors. Valuable phytochemicals from several plants have been in production using hairy root culture technology (Pedreño and Almagro, 2020; Rat et al., 2021; Roy, 2021). Hence, several researchers have put their efforts in development of hairy root cultures of plants with significance, using various strains of wild as well as modified *Agrobacterium rhizogenes*. Different methods of transformation of *O. sanctum* and *O. basilicum* using different media, antibiotics, and elicitors treatments have been described in Figure 5. Produced hairy roots were analyzed for different aspects like biomass, genetic transformation, and phytochemical and medicinal behavior, as described below:

### Bacterial Strain Used for Transformation

Strain ATCC 15834 (Tada et al., 1996; Bais et al., 2002; Vyas and Mukhopadhyay, 2014) and strain LBA 9402 (Skorupinska-Tudek et al., 2007; Marzouk, 2009) of *A. rhizogenes* were preferred during most studies to infect *O. sanctum* and *O. basilicum*. Other strains of *A. rhizogenes* used were MAFF 03-01724 (Tada et al., 1996), A4, Arqua1-pTSC5, 8196, and 11325 (Srivastava et al., 2016b, c).

Studies suggest that strains A-4, ATCC 15834, and LBA 9402 of *A. rhizogenes* were preferred to induce hairy roots as strains Arqua1-pTSC5, 8196, and 11325 were least effective to induce hairy roots from *Ocimum*.

### Media and Explant Used for Transformation

Mostly, MS media with or without agar was used for hairy root culture of *O. basilicum* and *O. sanctum*. However, G5 and WP mediums were also tried to culture hairy roots by Tada et al. (1996) and Skorupinska-Tudek et al. (2007). The use of claforan and cefotaxime is recommended, as these successfully cleared bacterial infection after co-cultivation (in the dark for 2 days). Only Marzouk (2009) used ampicillin for the same.

Mostly, young leaves of *in vitro*-germinated seedlings were used to infect with *A. rhizogenes*. Studies also reported the use of stems (Bais et al., 2002; Marzouk, 2009), as well as hypocotyl and cotyledon (Vyas and Mukhopadhyay, 2014; Srivastava et al., 2016b), for transformation purposes. Vyas and Mukhopadhyay (2014) studied green and red varieties of *O. sanctum*, and (Srivastava et al., 2016b, c) analyzed three cultivars of *O. basilicum* (Subja, Holy Green and Red Rubin) for development of hairy root cultures.

Therefore, leaves from green variety of *O. sanctum* and Subja cultivar of *O. basilicum* are better to achieve more clones and higher biomass on MS media after treatment of cefotaxime or claforan.

### Transformation Frequency of Hairy Roots

Leaves of green variety of *Ocimum sanctum* have been reported with maximum transformation frequency of 70%, followed by excised shoots of green form and red form (58 and 43%, respectively). Leaves of red form showed transformation frequency of 42%. Lowest transformation frequency of 26 and 21% was observed with hypocotyl of both red and green forms, respectively (Vyas and Mukhopadhyay, 2014).

Earliest root induction was observed with *A. rhizogenes* strain A4 in Subja cultivar (7 days), followed by Holy Green (10 days) and Red Rubin (13 days) cultivar. Late root induction (15 days) with a low survival rate in all cultivars was observed with *A. rhizogenes* strains ARqua1-pTSC5 and 11325, while strain 8196 was unable to induce root in any tissue. Maximum transformation efficiency of 70% was observed with leaves of Subja cultivar of *O. basilicum* (Srivastava et al., 2016b).

### Morphology of Hairy Roots

Insertion, copy number, and position of left and right fragments of T-DNA influence morphological assortment among hairy roots of different varieties, which determine phytochemical characteristic of hairy roots. Hairy roots from two varieties (red and green) of *O. sanctum* were mostly unbranched and densely covered with root hairs. Leaves of green variety produced higher number of hairy roots due to more phenylpropenes (signaling phenolics) as compared to red variety with more anthocyanins (Vyas and Mukhopadhyay, 2014).

Hairy roots of all the cultivars were observed with different characteristics. Subja cultivar was observed with maximum thickness (0.45 mm), Holy Green was observed with maximum number of root tips (1,317), and Red Rubin with maximum root length (1,112 cm) after 60 days of culture (Srivastava et al., 2016b). Microscopic analysis revealed no significant difference in root morphology of *O. basilicum*, even after 90 days of co-culture with *R. irregularis* (Srivastava et al., 2016c). All co-cultured hairy roots, as well as roots of seedlings, were observed with hyphae (intra- and extra-radical), arbuscules (active and collapsed), vesicles, and extra-radical spores. Maximum hyphal spread (dense) was observed with hairy roots of Red Rubin cultivar during the pre-symbiotic phase (20 days). During the symbiotic phase (after 40 days), hairy roots of Subja cultivar were observed with highest extraradical sporulation, as well as root length colonization percentage (32%) of *R. irregularis*. Therefore, co-cultivation with *R. irregularis* enhanced the overall characteristics and biomass of hairy roots in all cultivars of *O. basilicum*.

### Enhanced Biomass and Root Induction

Tada et al. (1996) and Bais et al. (2002) recorded maximum biomass when hairy roots of *O. basilicum* were infected with strain ATCC 15834 of *A. rhizogenes.* Tada et al. (1996) observed rapid proliferation in hairy roots of *O. basilicum* in WP and MS mediums after 3 weeks. Highest calculated biomass was 14.06 % DW (inoculum weight: 0.2-g FW) after 6 weeks (Tada et al., 1996) and 65 ± 5-g/l FW (inoculum weight: 100-mg FW) after 4 weeks (Bais et al., 2002) in MS medium.

Hairy root cultures of *O. basilicum* were subjected to treatments of salicylic acid (SA), jasmonic acid (JA), chitosan, and fungal cell wall elicitors (CWE) from *Phytophthora cinnamon* and *Phytophthora drechsleri* (Bais et al., 2002). Higher biomass of 65 ± 5-g/l FW (6.51-fold) of hairy roots (from 100-mg FW) could be further enhanced by 1.28-fold (up to 105.65 ± 7.9-g/l FW from 100-mg FW) using elicitors treatment (CWE). Although reduced biomass (10-g/l FW) was observed with other elicitors like SA, JA, and chitosan (Bais et al., 2002).

Similarly, when started with 3–4 hairy root tips to compare biomass among three cultivars (Subja, Holy Green, and Red Rubin) of *O. basilicum*, time-dependent increase was observed from 20 days (4–27-mg DW) to 60 days (59–112-mg DW) of culture. Subja and Holy Green cultivars accounted for highest biomass of 114- and 116-mg DW, respectively, as compared with 59-mg DW of Red Rubin (Srivastava et al., 2016b).

Lately, hairy root culture has been major focus to explore many plants. Hairy roots can be the best source of the desired metabolite and do not require growth regulators for culture. These studies demonstrated highly productive nature of hairy root cultures of pharmaceutically vital plant like *Ocimum* to enhance biomass, which ultimately resulted in heightened phytoceuticals for the therapeutic industry.

## Phytochemical Investigation

Accumulated metabolites directly reflect the commercial importance of all plant parts, including roots, of medicinal plants. A number of studies mentioned phytochemical profile of leaves and seeds of *Ocimum sp*., but revelation of metabolic composition of roots remained understated. Ahmad et al. (2012a; 2012b) and Kumar et al. (2015) analyzed natural root tissues of field-grown *O. sanctum*, while hairy roots of *O. sanctum* (Skorupinska-Tudek et al., 2007) and *O. basilicum* (Tada et al., 1996; Bais et al., 2002; Marzouk, 2009) were mostly studied from methanolic root extracts for phytochemicals due to their commercial application. Solvent used for extraction of diverse explants of varied culture conditions to isolate and identify different metabolites has been mentioned in Table 3.

**TABLE 3:** Identification of phytochemicals from soil-grown, *in vitro*, and hairy roots of *Ocimum basilicum* and *Ocimum sanctum*.

Plant	Explant used	Treatment	Extraction	Analytical method	Metabolites analyzed/identified	References
*O. sanctum*	*In vitro* roots; soil grown roots	WP medium	acetone/hexane (1:1, v/v)	TLC, HPLC	Isoprenoid alcohols (10.6 μg/g DW)	Skorupinska-Tudek et al., 2007
	Roots	Filed soil	MeOH	TLC, Column chromatography, NMR	urs-12-en-3β,6β,20β-triol-28-oic acid^#^; 1″-menthyl-2-glucopyranosyloxybenzoate^#^; n-decanoyl-β-D-glucopyranosyl-(2a→1b)-β-D-glucopyranosyl-(2b→1c)-β-D-glucopyranosyl-(2c→1d)-β-D-glucopyranosyl-2d-2′-hydroxybenzoate^#^; ursolic acid^#^; palmityl glucoside^#^	Ahmad et al., 2012a
			MeOH	TLC, Column chromatography, NMR	stigmast-5-en-3b-olyl-n-eicos-9-12-dienoate^#^; n-octacos-9-enoic acid^#^; lup-12, 20(29)-dien-28-oic-acid-3b-olyl-octadecanoate^#^; ursolic acid^#^; 3-epibetulinic acid^#^	Ahmad et al., 2012b
			Ethyl acetate	HPLC	Flavonoids: flavones and flavonols^#^	Kumar et al., 2015
*O. basilicum*	Hairy roots	MS, B5, WP liquid media	MeOH	HPLC	Rosmarinic acid (73.5 mg/flask); Lithospermic acid A (1.70% DW); Lithospermic acid B (0.17% DW)	Tada et al., 1996
		MS media; SA, JA, chitosan, fungal CWE (*Phytophthora cinnamon* and *P. drechsleri*)	MeOH	HPLC, NMR	Rosmarinic acid (143.8 ± 7.3 g/L); Lithospermic acid B^#^	Bais et al., 2002
		MS media	MeOH, CH_2_Cl_2_	TLC, Column chromatography, NMR	**Betulinic acid (37 mg); **3-epimaslinic acid (34 mg); **Alphitolic acid (105 mg); **Euscaphic acids (54 mg); **Oleanolic acid (4 mg); **Ursolic acid (6 mg)	Marzouk, 2009
*O. basilicum* (3-cultivars)	Leaves, flowers, roots	Pot soil	MeOH	Folin’s test; HPLC-coupled acidic potassium permanganate chemiluminescence assay	Total phenolics (66–86 mg/g DW); Chicoric acid (0.2–1.6 mM/100 g DW); Rosmarinic acid (4–14 mM/100 g DW); 3-hydroxybenzoic acid^#^; m-coumaric acid^#^; p-coumaric acid^#^; caffeic acid^#^; ferulic acid^#^; Vanillic acid^#^	Srivastava et al., 2016a
	Hairy roots	MS media			Total phenolics (up to 378.8 mg/g DW); Rosmarinic acid (up to 76.4 mg/g DW); Caffeic acid (up to 1.74 mg/g DW)	Srivastava et al., 2016b
		*Rhizophagus irregularis*			Total phenolics (101–425 mg/g DW); Rosmarinic acid (14–150 mg/g DW); Caffeic acid (0.3–2.46 mg/g DW)	Srivastava et al., 2016c
*O. sanctum*, *O. kilimandscharicum*	Hairy roots	Field soil	MeOH, C_6_H_14_, CHCl_3_, EtAc, C_4_H_9_OH, H_2_O	Spectrophotometer, HPLC	Total flavonoids; Total phenolics (3.46 ± 0.49 to 29.88 ± 2.89 μg/mg DW); Rosmarinic acid^#^; Caffeic acid^#^; Hydroxyl cinnamic acid^#^; Caffeoyl dihydroxy phenyl lactoyl- tartaric acid^#^	Agarwal et al., 2017

*^#^Quantity not available; ^∗∗^per 273.5-g FW;*

*MS, Murashige and Skoog; WP, woody plant; B5, Gamborg B5; SA, salicylic acid; JA, jasmonic acid; CWE, cell wall elicitors; RA, rosmarinic acid; TLC, thin layer chromatography; HPLC, high-performance liquid chromatography; GC-MS, gas chromatography-mass spectrometry; GC-FID, gas chromatography-flame-ionization detection; NMR, nuclear magnetic resonance; FAAS, flame atomic absorption spectroscopy; ICP-OES/MS, inductively coupled plasmaoptical emission/mass spectrometry; MeOH, methanolic water; CH_2_Cl_2_, dichloromethane; C_6_H_14_, hexane; CHCl_3_, chloroform; EtAc, ethyl acetate; C_4_H_9_OH, butanol; H_2_O, water; RE, root extract; DW, dry weight.

### Rosmarinic Acid and Phenolics: Due to Different Bacterial Strains and Media Composition

Roots of *Ocimum* accumulate rosmarinic acid (RA) as the principal antioxidant phenolic compound, as compared to other tissues of the plants (Bais et al., 2002). Secondary metabolite content, which varies in hairy roots based on different culture conditions (like *Agrobacterium* strain, media composition, and elicitors treatment), is discussed below:

Hairy roots of *O. basilicum* obtained after infection of *A. rhizogenes* (strains ATCC 15834 and MAFF 03-01724) were cultured on three different types of liquid media (MS, B5, and WP). Methanolic extract of lyophilized hairy roots was analyzed through HPLC for amount of rosmarinic acid (RA) and related phenolics (lithospermic acids A and B). Higher concentration of RA was obtained from hairy root culture obtained from infection of MAFF 03-01724 in MS media (14.1%), as well as from infection of ATCC 15834 strain on B5 media (14%). Hairy roots cultured on MS media after infection of *A. rhizogenes* strain MAFF 03-01724 accumulated up to 73.5 mg/flask of RA (maximum). Lithospermic acid A was observed maximum (1.7%) in hairy root clones with MAFF 03-01724 infection, cultured in MS media. Accumulation of lithospermic acid B (0.17%) was observed in B5 and WP media (Tada et al., 1996).

Hairy roots of *O. basilicum* obtained using strain MAFF 03-01724 produced a higher level of rosmarinic acid and lithospermic acid A in the presence of MS media. However, when the media was replaced with B5 and WP, a greater number of phenolics were extracted. Optimization of macronutrients has benefits in root growth (Arab et al., 2018) and *Agrobacterium*-mediated transformation (Hata et al., 2021). Therefore, selection of effective media can be very advantageous to attain the desired trait in *Ocimum*.

### Difference in Rosmarinic Acid Due to Elicitors Treatments

Hairy roots of *Ocimum basilicum* obtained from infection of *A. rhizogenes* (ATCC 15834) were analyzed for RA with and without elicitor(s) treatment. Chitosan SA, JA, and fungal CWE (*P. cinnamon* and *P. drechsleri*) were used to treat hairy root cultures. HPLC revealed maximum accumulation of RA in hairy roots as compared to leaves, shoots, and roots of *in vitro*-grown plants. Hairy roots accumulated 2.98%-g-FW-higher RA, as compared to non-transformed roots. Elicitation due to CWE further enhanced RA content by 8.1 ± 0.59%-g FW on 28th day of culture. Specifically, CWE of *P. cinnamon* and *P. drechsleri* increased concentration of RA (in dose-dependent manner) on 12th day by 1.54-fold (125.8 ± 9.4 g/l) and 1.64-fold (143.8 ± 7.3 g/l), respectively (Bais et al., 2002).

Thus, results indicate that accumulation of RA in root tissue of *O. basilicum* can be increased by synergistic effect of transformation and elicitors.

### Isoprenoids and Triterpenes in Hairy Roots

Extracts (lipid fractions) of hairy roots, roots, stems, and leaves of *O. sanctum* were analyzed for polyisoprenoid by Skorupinska-Tudek et al. (2007) and compared with extracts of few more plants (TLC and HPLC). Total isoprenoid content was lowest in root tissues (2–6-μg/g DW) of *O. sanctum* followed by stem (3–9-μg/g DW) and leaves (77–100-μg/g DW). This is because the photosynthetic tissues accumulate higher amounts of polyprenols in the presence of sunlight. Hairy roots of *O. sanctum* grown in WP media showed significant quantity of isoprenoids (up to 15-μg/g DW). Therefore, even the metabolite with lower concentration in normal roots of *O. sanctum* can be extracted in higher amount using hairy root culture.

Marzouk (2009) presented similar a TLC-screening pattern of triterpenes in normal and hairy roots (273.5-g FW); however, 3-fold more extractives were collected from a hairy root of *O. basilicum* (4 weeks old). NMR analysis of these purified extracts has led to isolation and identification of six major triterpenes. These triterpenes were betulinic acid (37 mg), 3-epimaslinic acid (34 mg), alphitolic acid (105 mg), euscaphic acids (54 mg), oleanolic acid (4 mg), and ursolic acid (6 mg). Among these oleanolic acid, 3-epimaslinic acid and euscaphic acids were reported for the first time from *O. basilicum*. All the identified terpenoids proved to be hepatoprotective in nature.

### Phytochemical Difference Among Cultivars

Three cultivars (Subja, Holy Green, and Red Rubin) of *O. basilicum* were studied for difference in polyphenolics (3-hydroxybenzoic, m-coumaric, p-coumaric, caffeic, ferulic, vanillic, chicoric, and rosmarinic acids) by Srivastava et al. (2016a, b, c). Ethanolic extract of different plant parts of 90-day-old plants (Srivastava et al., 2016a), hairy roots (Srivastava et al., 2016b), and co-cultured hairy roots with 75-day-old spores of *Rhizophagus irregularis* were quantified using HPLC (Srivastava et al., 2016c).

Highest accumulation of rosmarinic acid and chicoric acid was reported in leaves and roots of Holy Green. Flowers of Subja gave higher and the Red Rubin showed undetected levels of chicoric acid (Srivastava et al., 2016a). Maximum accumulation of RA experienced in hairy roots at 40, 50, and 60 days in Subja (71.03 ± 12.67-mg/g DW), Holy green (69.49 ± 1.79-mg/g DW), and Red Rubin (76.41 ± 3.71-mg/g DW) cultivars, respectively. Subja cultivar showed 5-fold-higher accumulation of RA in hairy roots as compared to non-transformed roots. Among all cultivars, hairy roots of Holy green were detected with maximum CA accumulation (1.74–0.17-mg/g DW) at 50 days. Although Subja cultivars accumulated highest amount of RA till 40 days, Holy green cultivar can be better to maximize accumulation of both RA and CA till 50 days of culture (Srivastava et al., 2016b). Colonization with *R. irregularis* influenced concentration of RA and CA in hairy roots (Srivastava et al., 2016c). Colonized hairy roots of Subja, Holy Green, and Red Rubin cultivars observed with maximum 1.9-fold (60 days), 2.1-fold (30 days), and 1.6-fold (30 days) increase in RA content, respectively. Co-culture with *R. irregularis* improved accumulation of CA in 30-day-old hairy roots of Subja (6.7-fold), followed by Holy Green (3.3-fold) and Red Rubin (1.3-fold).

The leaves of all three cultivars were detected with similar (66.99 to 85.53-mg/g DW) total phenolic content. Subja cultivar was observed with significantly higher total phenolic content in flowers (68.20 ± 5.54 mg/g DW), roots (80.69 ± 4.58-mg/g DW) and the whole plant (219.22 ± 4.50-mg/g DW) when compared with other cultivars of *O. basilicum* (Srivastava et al., 2016a). Hairy roots of Holy green cultivars were calculated for maximum amount (139.1–378.8-mg/g DW) of total phenolics from 20 to 60 days of culture, followed by hairy roots of Subja cultivar (127.7–354.9-mg/g DW). Total phenolics in cultivar Red Rubin increased slowly till 40 days, which enhanced significantly from 161.6 to 374.9-mg/g DW from 40 to 60 days of culture (Srivastava et al., 2016b). Total phenolic content of Subja and Red Rubin hairy roots was enhanced by 44–50-mg/g DW at 60 days of co-culture with *R. irregularis* (Srivastava et al., 2016c). More endogenous IAA (up to 0.039 μg/mg) was quantified using HPLC in hairy roots of all cultivar of *O. basilicum*, although content of IAA varies among different clones of same cultivars (Srivastava et al., 2016b).

Srivastava et al. (2016a) experienced highest antioxidant potential in roots of Subja and leaves of other cultivars. However, flowers of Subja were observed with greater antioxidant potential as compared to flowers of Holy Green and Red Rubin. Srivastava et al. (2016b) stated time-dependent increase (2-fold) in total antioxidant potential with no significant difference among hairy roots of three cultivars. Srivastava et al. (2016c) boosted antioxidant potential up to 4-, 2-, and 5-fold for total phenolics as well as 5-, 4-, and 12-fold for RA in hairy roots of Subja, Holy Green, and Red Rubin cultivars after colonization with *R. irregularis*.

Thus, it can be concluded that rosmarinic acid is major antioxidant in all cultivars of *O. basilicum*. Caffeic acid (CA) was also spotted as a minor metabolite in hairy roots of *O. basilicum.* Time-dependent increased accumulation of these metabolites and total phenolics was achieved through hairy root culture in all three cultivars. Increase in antioxidant potential from normal roots to transformed hairy roots and elicited hairy roots (with *R. irregularis*) represent enhanced metabolic composition of culture. Although roots and leaves of Holy green cultivar accumulated maximum phenolics, including RA, hairy roots of Subja cultivar could be considered best for extraction due to its morphology with enhanced biomass, antioxidant potential, total phenolics, and RA (40 days old).

### Metabolites in Roots of Field-Grown Plants

Ahmad et al. (2012a) analyzed methanolic roots extract of *O. sanctum* (2.5 kg) for isolation of three new phytoconstituents. These compounds were identified as stigmast-5-en-3b-olyl-n-eicos-9-12-dienoate, n-octacos-9-enoic acid, and lup-12, 20(29)-dien-28-oic-acid-3b-olyl-octadecanoate, along with known compounds of ursolic acid and 3-epibetulinic acid.

Another study of methanolic root extracts of field-grown *O. sanctum* by Ahmad et al. (2012b) identified five phytochemicals using nuclear magnetic resonance (NMR), fast atom bombardment (FAB) mass, ^1^H-^1^H correlation spectroscopy (COSY), and heteronuclear multiple-bond correlation (HMBC) spectral techniques. Two compounds, ursolic acid and palmityl glucoside, have already been reported. Three unknown identified compounds were called as urs-12-en-3β,6β,20β-triol-28-oic acid, 1″-menthyl-2-glucopyranosyloxybenzoate, and n-decanoyl-β-D-glucopyranosyl-(2a→1b)-β-D-glucopyranosyl-(2b→1c)-β-D-glucopyranosyl-(2c→1d)-β-D-glucopyranosyl-2d-2′-hydroxybenzoate.

Kumar et al. (2015) analyzed different fractions (hexane, chloroform, ethyl acetate, and butanol) of root methanolic extracts from field-grown *O. sanctum*. These extracts were monitored using high-performance liquid chromatography (HPLC) fingerprints to categorize flavonoids (flavones, flavonols, and dihydroflavonols), including glycone and aglycones. Kumar et al. (2015) provided preliminary phytochemical screening as chromophoric groups and did not quantify the metabolites.

Improvement in metabolic awareness of *Ocimum* roots generates more possibilities to explore and utilize roots as starting material. *In vitro*-established root cultures are best to harvest desired product using the best possible combination for maximum accumulation of phytoceuticals present.

## Conclusion

Promising therapeutic activities, like hepatoprotective, antimicrobial, antimalarial, and antidiabetic, are associated with *Ocimum* roots. Although antimicrobial nature of *Ocimum* roots protects from several pathogens, conversely, microbes of rhizosphere also affect the roots in both ways. Chemical nature of soil, soil treatments (fungicide and pesticides), as well as microbes and contaminations (radioactive, salt, and heavy metal), define the suitability of soil in negative or positive manner. Hence, rhizosphere is very crucial as it furnishes supplements to signals *via* roots to the whole plant. Effect of different rhizospheric elements on roots, as well as whole plants, provides the better understanding of required eco-friendly and biosafe procedure for industries, including agriculture and bioprocess industries.

*In vitro* techniques stand best among such procedures, which include adventitious and hairy root cultures as promising candidates to support the phytochemical industry by reducing manpower and duration of field cultivation. *Ocimum* roots being very useful due to significant amount and variety of phytoceuticals were thoroughly studied. Improved biomass and quantity of phytoceuticals were achieved through adventitious or hairy root cultures of different species as well as cultivars of *Ocimum*. Phytochemical profile of roots with different treatments simplifies higher production of phytoceuticals.

## Author Contributions

VP and RS has drafted the review under the guidance of AN and written the review article. AN has further critically read the article. All authors contributed to the article and approved the submitted version.

## Conflict of Interest

The authors declare that the research was conducted in the absence of any commercial or financial relationships that could be construed as a potential conflict of interest.

## Publisher’s Note

All claims expressed in this article are solely those of the authors and do not necessarily represent those of their affiliated organizations, or those of the publisher, the editors and the reviewers. Any product that may be evaluated in this article, or claim that may be made by its manufacturer, is not guaranteed or endorsed by the publisher.

## Abbreviations

MS: Murashige and Skoog
WP: woody plant
B5: Gamborg B5
FW: fresh weight
DW: dry weight
NAA: α-naphthaleneacetic acid
Kn: Kinetin
2, 4-D: 2,4-dichlorophenoxyacetic acid
BAP: 6-benzylaminopurine
PGR: plant growth regulator
DPPH, 2: 2-Diphenyl-1-picrylhydrazyl
NRA: nitrate reductase activity
NP-SH: nonprotein thiol
MDA: malondialdehyde
CAT: catalase
APX: ascorbate peroxidase
GPX: guaiacol peroxidase
SOD: superoxide dismutase
TLC: thin layer chromatography
HPLC: high-performance liquid chromatography
GC-MS: gas chromatography-mass spectrometry
GC-FID: gas chromatography-flame-ionization detection
NMR: nuclear magnetic resonance
FAAS: flame atomic absorption spectroscopy
ICP-OES/MS: inductively coupled plasmaoptical emission spectrometry/mass spectrometry
SA: salicylic acid
JA: jasmonic acid
CWE: cell wall elicitors
RA: rosmarinic acid
K: potassium
CFU: colony-forming unit
PFU: plaque-forming unit.
